# Foundation Models for Histopathology—Fanfare or Flair

**DOI:** 10.1016/j.mcpdig.2024.02.003

**Published:** 2024-03-05

**Authors:** Saghir Alfasly, Peyman Nejat, Sobhan Hemati, Jibran Khan, Isaiah Lahr, Areej Alsaafin, Abubakr Shafique, Nneka Comfere, Dennis Murphree, Chady Meroueh, Saba Yasir, Aaron Mangold, Lisa Boardman, Vijay H. Shah, Joaquin J. Garcia, H.R. Tizhoosh

**Affiliations:** a KIMIA Lab, Department of Artificial Intelligence & Informatics, Mayo Clinic, Rochester, MN; b Department of Dermatology, Mayo Clinic, Rochester, MN; c Department of Laboratory Medicine and Pathology, Mayo Clinic, Rochester, MN; d Comprehensive Cancer Center, Mayo Clinic, Rochester, MN; e Department of Dermatology, Mayo Clinic, Phoenix, AZ

## Abstract

**Objective:**

To assess the performance of the current foundation models in histopathology.

**Patients and Methods:**

The assessment involves a comprehensive evaluation of some foundation models, such as the CLIP derivatives, namely PLIP and BiomedCLIP, which were fine-tuned on data scraped from the internet. The comparison is performed against simpler and nonfoundational histology models that are trained on well-curated data, eg, the cancer genome atlas. All models are evaluated on 8 datasets, 4 of which are internal histology datasets collected and curated at Mayo Clinic, and 4 well-known public datasets: PANDA, BRACS, CAMELYON16, and DigestPath. Evaluation metrics include accuracy and macro-averaged F1 score, using a majority vote among top-k (eg, MV@5) at the whole slide image/patch levels. Moreover, all models are evaluated in classification settings. This detailed analysis allows for a deep understanding of each model’s performance across various datasets.

**Results:**

In various evaluation tasks, domain-specific (and nonfoundational) models like DinoSSLPath and KimiaNet outperform general-purpose foundation models. The DinoSSLPath excels in whole slide image-level retrieval for internal colorectal cancer and liver datasets with MV@5 macro-averaged F1 scores of 63% and 74%, respectively. The KimiaNet leads in breast and skin cancer tasks with respective Top-1 and MV@5 scores of 56% and 70%, respectively and scores 75% on the public CAMELYON16 dataset. Similar trends are observed in patch-level metrics, highlighting the advantage of using specialized datasets like the cancer genome atlas for histopathological analysis.

**Conclusion:**

To enable effective vision-language foundation models in biomedicine, high-quality, multi-modal medical datasets are essential. These datasets serve as the substrate for training models capable of translating research into clinical practice. Of importance, the alignment (correspondence) between textual and visual data—often diagnostic—is critical and requires validation by domain experts. Thus, advancing foundation models in this field necessitates collaborative efforts in data curation and validation.

Foundation models in artificial intelligence are considerably affecting various domains, including natural language processing^1^, ^2^, ^3^ and computer vision.^4^^,^^5^ These models excel at learning general features from vast datasets, allowing for targeted fine-tuning for domain-specific tasks.^6^^,^^7^

In biomedicine, specialized language models such as BioBERT and BioGPT have shown promise, largely owing to unsupervised training on extensive datasets.^8^, ^9^, ^10^, ^11^ Such models obviate the need for data annotation and outperform their general-purpose counterparts. Alternatively, CLIP serves as another class of foundation models, distinctively facilitating the integration of visual and textual data. With training on a corpus of 400 million image-text pairs, it bridges the semantic disconnect between these 2 domains, offering wide-ranging applications. However, the task of precise vision-language correspondence (alignment) poses considerable challenges, especially in the specialized biomedical realm (Figure 1).

**Figure 1:**
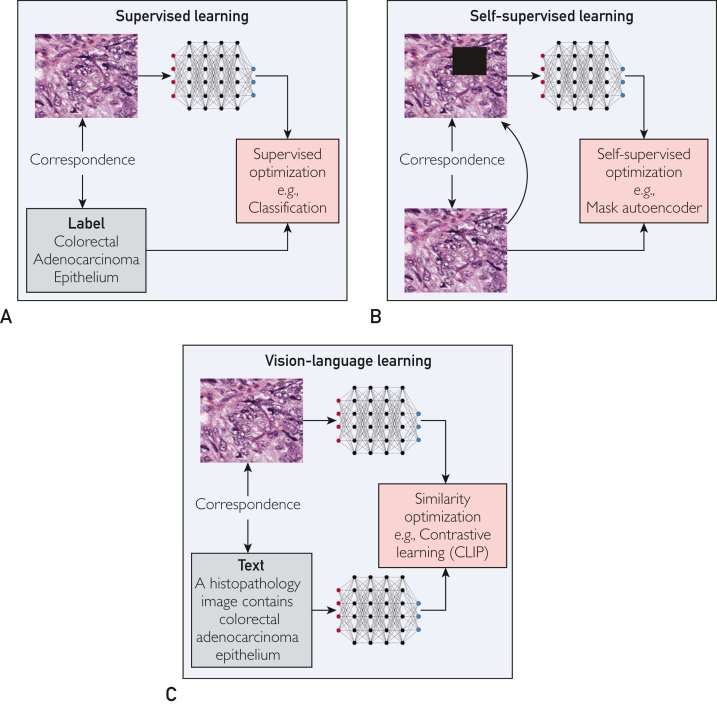
Illustration of 3 deep learning methodologies in medical imaging. A, In supervised learning, meticulous data curation, and human annotation are crucial. This approach uses both the input data and its corresponding label for model fine-tuning. Integrity is maintained through rigorous human review. B, Self-supervised learning eliminates the need for human annotation by automatically generating correspondences between the original and augmented or masked images. C, Image-text learning methods, like contrastive algorithms such as CLIP, require human validation to ensure accurate image-text alignment. This is particularly critical in medical settings, where expert consensus, often from pathologists, is essential for validation.

In the specific area of histology image analysis, foundation models such as PLIP^12^ and BiomedCLIP^13^ have not matched the performance of specialized models like KimiaNet^14^ and DinoSSLPath^15^, both trained on high-quality, domain-specific datasets. The limited scale of training data for PLIP and BiomedCLIP contributes to this performance gap.

The development of foundation models for biomedical applications presents challenges, especially concerning the need for high-quality, expert-validated data. In histopathology, for instance, images possess unique attributes such as limited color diversity and high-resolution cellular structures, complicating model training. A focused approach involving expert-validated, domain-specific datasets is essential for crafting reliable foundation models crucial for advancing clinical and research applications.

## Methods

### Study Design

We performed a comprehensive analysis on a diverse collection of histopathological image datasets from both private and public sources. The study followed 4 main phases, as follows: data acquisition, model selection, evaluation, and interpretation of results. Our primary data source was a set of 4 internally curated Mayo datasets, comprising various anatomical sites and pathological conditions.

In model selection because there are some internet-sourced vision-language models, such as CONCH^16^ that is not publicly available, we opted for a range of the recent publicly available ones, eg, PLIP and BiomedCLIP, to ensure architectural and domain diversity. Specifically, DinoV1 and DinoSSLPath use transformer architectures and self-supervised learning, whereas BiomedCLIP and PLIP deploy contrastive learning on medical image-text pairs. Conventionally trained models with public data, ie, KimiaNet and DinoSSLPath, have also been included.

**Figure 2:**
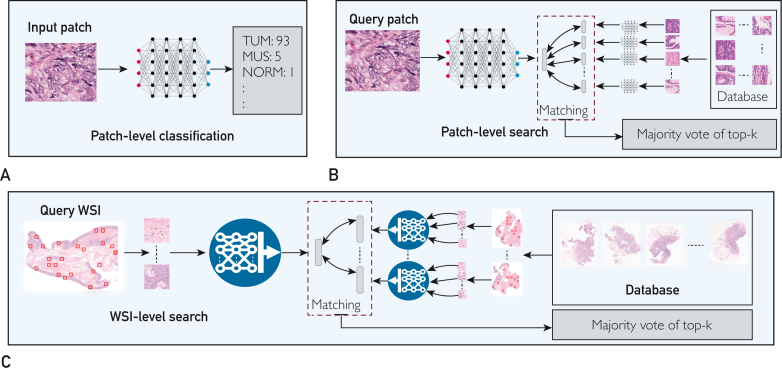
Histopathological image analysis tasks. A, The patch-level classification task involves the categorization of distinct histological patches, extracted from their Whole Slide Images (WSIs). B, The patch-level retrieval task entails the comparison of embeddings from a query histology patch against a large database of such embeddings to retrieve the most similar instances. C, In the WSI-level retrieval task, the embedding of a query WSI is compared with the embeddings of all WSIs stored in the database, aiming to identify and retrieve the most similar WSIs. Subsequent to the retrieval process, a majority vote is computed from the top-k retrieved instances to recommend a label of the input patch or WSI.

The evaluation phase involved a comprehensive set of experiments for 3 tasks, as follows: patient-level search, patch-level search, and image classification. Figure 2 depicts these 3 histology image analysis tasks. Validation was performed internally on the Mayo datasets across 4 different sites (breast, skin, colorectal, and liver) and externally on publicly available datasets like PANDA,^17^ CAMELYON16,^18^ and BRACS.^19^ Model performances were systematically contrasted with existing benchmarks in histopathological image analysis.

### Data Source

Our study used 8 datasets, including 4 internally curated Mayo datasets from Mayo Clinic. These whole slide image (WSI) sets cover breast, liver, colorectal cancer (CRC), and skin, each capturing a range morphology for relevant pathological conditions.

The Mayo skin dataset includes 660 WSIs from patients diagnosed with cutaneous squamous cell carcinoma, featuring 386 well-differentiated, 100 moderately differentiated, and 67 poorly differentiated patients, and 107 patients with normal skin WSIs. Patient demographics indicate a median age of 77, with females constituting 35% of cases. The liver dataset comprises 150 WSIs from alcoholic steatohepatitis (ASH) cases and 158 from nonalcoholic steatohepatitis (NASH) cases, sourced primarily from liver biopsies. The ASH diagnoses are clinician-reviewed, whereas NASH cases involve obese patients undergoing bariatric surgery. The CRC dataset includes 209 WSIs categorized into cancer adjacent polyp, nonrecurrent polyp, and recurrent polyp. The breast dataset consists of 73 WSIs classified into 16 tumor subtypes, encapsulating diverse pathologies like, among others, adenoid cystic carcinoma, ductal carcinoma in situ, and invasive breast carcinoma of no special type. We do not have specific exclusionary or inclusionary criteria for time, but some samples are from as far back as 1996.

To broaden the scope of our analysis, we used publicly available WSI datasets, namely, Prostate cANcer graDe Assessment (PANDA),^17^ BReAst Carcinoma Subtyping (BRACS),^19^ and CAMELYON16.^18^ In addition, we incorporated patch-level dataset, namely DigestPath, thereby diversifying the histological domains under consideration.

PANDA dataset^17^ consisted of 12*,*625 WSIs of hematoxylin and eosin-stained prostate biopsies including 10*,*616 biopsies for development, 393 for tuning, 545 for internal validation, and 1071 for external validation collected from 6 sites in The Netherlands, Sweden, and the United States. The CAMELYON16 dataset^18^ contains 399 WSIs of lymph node sections from breast cancer patients. The slides were collected from 2 hospitals in the Netherlands and meticulously annotated for metastases under pathologist supervision, using immunohistochemistry when needed. The slides containes macrometastases, micrometastases or isolated tumor cells. We used the test set of CAMELYON16 which contains 129 WSIs. The BRACS dataset^19^ contains 547 WSIs from 189 patients. The images were scanned at 0.25 μm/pixel magnification. The lesions were annotated by 3 board-certified pathologists into 7 sub- types, as follows: normal (484 RoIs), pathological benign (836 RoIs), usual ductal hyperplasia (517 RoIs), flat epithelial atypia (756 RoIs), atypical ductal hyperplasia (507 RoIs), ductal carcinoma in situ (790 RoIs), and invasive carcinoma (649 RoIs). We used the entire dataset since we apply leave-one-out validation. Overall, we used the WSI-level for the patient-level retrieval, and we used ROIs for patch-level retrieval and classification.

DigestPath dataset^20^, developed in collaboration with Chinese medical institutions, comprises 2 subdivisions for gastrointestinal pathology: signet ring cell detection dataset and colonoscopy tissue segmentation and classification dataset. This resource covers patient ages from 20-70 years old and maintains gender equilibrium. Histological slides are stained using hematoxylin and eosin (H&E) and captured with a KFBIO FK-Pro-120 scanner, with expert pathologist annotations.

The combined use of these diversified datasets facilitates a comprehensive and nuanced evaluation of foundation models (FMs) in histopathological image analysis. These datasets provide a variety of histological images from different anatomical sites and pathological conditions and represent images of different patient populations captures by different machines. As such they aid to establish a deeper, more insightful understanding of the capabilities and limitations inherent to the evaluated foundation and ordinary models.

### Procedures

We evaluated a diverse array of deep learning models, specifically PLIP, BiomedCLIP, DinoV2, CLIP, KimiaNet, and DinoSSLPath. Our evaluation methodology targeted 3 key histology image analysis tasks: patient-level retrieval, patch-level retrieval, and patch classification. In essence, the search (retrieval) task is deemed more adept at evaluating the intrinsic quality of the backbone. To further appraise the classification performance while building on pre-extracted embeddings, we performed a patch-level 5-fold cross-validation, thereby elucidating the feature quality inherent in each backbone.

In the first task, we performed a patient-level search by leveraging the Yottixel framework. This framework allows for the matching of a query patient to similar cases within a comprehensive database, based on their histological characteristics. The patient query was constructed in a mosaic,^21^ a represented set of histology patches sampled from the entire WSI of that particular patient. We performed this evaluation on 4 internal datasets from Mayo Clinic —Skin, Breast, Liver, and CRC— and 3 publicly available WSI datasets, including PANDA, CAMELYON16, and BRACS. The performance metrics, specifically accuracy, and Macro F1-score, were calculated using the leave-one-patient-out validation approach. This enabled a nuanced evaluation of the ability of each model to accurately identify and retrieve similar patients based on their visual histopathological features.

For the second task, we aimed to rigorously examine the models’ embedding quality by a patch-level search task. Employing a leave-1-patient-out scheme, we excluded all patches from the same WSI as the query patch. Unlike patient-level retrieval, this task enabled micro-level performance evaluation, concentrating on the relevance of individual patches. All available datasets were used for this task. In the case of WSI-level datasets, we leveraged the Yottixel’s mosaic to select the most representative patches of a WSI in an unsupervised manner.

The final evaluation task is the patch-level classification, performed by a linear-prob classifier. We first extracted patch-level feature embeddings from each model for each dataset. After this, a simple linear classifier was trained for each dataset’s specific classes in a 5-fold cross-validation scheme. The evaluation for this task was performed on 4 internal and 4 public datasets. This extensive dataset inclusion served to enhance the depth and diversity of our analysis, providing a comprehensive view of each model’s performance across varied conditions and anatomical sites.

Through this rigorous evaluation protocol, we aimed to holistically understand the performance, strengths, and limitations of the vision-language FMs as compared with domain-specific models in histopathology image analysis.

### Outcomes

The primary outcome of our study centered on investigating the applicability and efficacy of FMs in real-world histopathological settings. Employing a rigorous evaluation method, incorporating both WSI and patch-level analyses, metrics such as Top-1, majority vote among top-3 (MV@3), and majority vote among top-5 (MV@5) revealed notable performance deficiencies in CLIP derivatives. These shortcomings may predominantly be attributed to data scale and data quality constraints. The multi- layered evaluation methodology encompassed WSI-level alignment through deep learning techniques and patch-level nearest-neighbor searches, concluding that the field currently lacks genuine vision-language FMs optimized for histological applications. The secondary outcome involved the assessment of model generalizability and downstream task efficacy without additional fine-tuning. This exhaustive benchmarking illuminated the inherent capabilities and limitations of each scrutinized model, highlighting the necessity for meticulous data curation and enhanced training paradigms. Specifically, leave-1-patient-out and 5-fold cross-validation strategies were employed for search (retrieval) and classification tasks, respectively. Resultant metrics, including accuracy (Acc) and macro-averaged F1 Score (Macro), were reported, alongside majority voting techniques MV@3 and MV@5, further substantiating the study’s comprehensive findings.

### Statistical Analyses

Experiments were performed under identical computational settings on a machine equipped with 4 × NVIDIA A100-SXM4-80GB GPUs, using PyTorch 2.0.0, CUDA 11.7, and Python 3.9.16. Six histology models—DinoV2-B-16, CLIP (ViT-B/32), BiomedCLIP (PubMedBERT-256-vit Base of patch 16), PLIP (ViT-B-32), DinoSSLPath (DINO patch 8), and KimiaNet were assessed with default input configurations.

For WSI-level evaluation, we adopted Yottixel’s general workflow for indexing and retrieval, with minor adjustments.^21^ The WSIs were patched into fixed-size patches (1000 × 1000 pixels at 20× magnification). During tissue segmentation, patches containing at least 70% tissue, measured by area, were retained, whereas the remainder were discarded as irrelevant background. To optimize computational efficiency, a representative patch subset—∼5% of each WSI—was selected through Yottixel’s mosaic, an RGB-based and proximity-based clustering followed by intra-cluster sampling.^21^ These subsets, called mosaics, were subsequently processed by deep networks to extract high-dimensional embeddings, serving as indexed WSI representations. One-to-1 Euclidean distances between patch embeddings were computed and their median employed as the distance metric for WSI similarity evaluation as proposed in literature.^21^^,^^22^ On internal datasets, the experiments were performed with 20X magnification images. For patch-level search on the WSI datasets, we used the patches selected by Yottixel and employed the leave-1-out evaluation. Finally, for the patch classification, we employed the linear classifier from Sklearn package (ie, SGD Classifier) for 10*,*000 iterations. In our evaluation, we retain the recommended input size for each model. This is crucial in deep learning, as consistency with the input data configurations used during training is essential for meaningful validation.

## Results

In this section, we report the performance of several models on histology image analysis including 4 FMs DinoV2^23^ (with ∼87 million parameters), CLIP^24^, BiomedCLIP^13^, and PLIP^12^ (all with ∼196 million parameters), and 2 nonfoundational models DinoSSLPath^15^ (with ∼22 million parameters), and KimiaNet^14^ (with ∼7 million parameters). Whereas PLIP has used 208,000 online histology images on top of 400 million CLIP images, DinoSSLPath has been trained with 19 million the cancer genome atlas (TCGA) patches whereas KimiaNet has only used 240,000 TCGA patches on top of 1.2 million DenseNet images.

**Figure 3:**
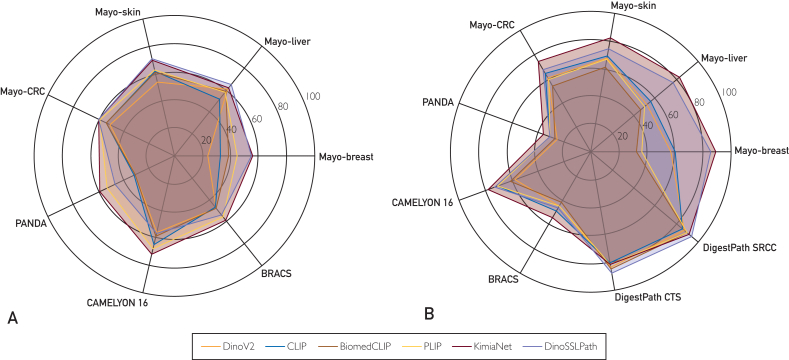
Model performance on public and private datasets shows: (A) top-1 macro F-score under WSI-level search and (B) macro F-score of 5-fold cross-validation. WSI, whole slide images.

In the WSI-level search, Table 1 reports the performance metrics of 6 histology image feature extractors across 4 internal and 3 public datasets. For the internal datasets, DinoSSLPath achieved the highest MV@5 macro-averaged F1 Score in the CRC and liver categories, registering 63% and 74%, respectively. The KimiaNet led in the breast and the skin categories with macro-averaged F1 Scores of Top-1 56% and mV@5 61%, respectively. On public datasets, KimiaNet provides the highest performance on the PANDA dataset with MV@5 macro-averaged F1 Score of 55%, and on the CAMELYON16 dataset with 75%. Figure 3(A) visualizes the Macro F-score performance.

**Table 1:** Patient-Matching Outcomes Across 4 Internal and 3 Public Datasets for Search (retrieval), Subtyping, and Grading tasks^a^

Dataset		DinoV2		CLIP		BiomedCLIP		PLIP		KimiaNet		DinoSSLPath	
		Acc	Macro	Acc	Macro	Acc	Macro	Acc	Macro	Acc	Macro	Acc	Macro
Internal Data													
Breast	Top 1	36%	24%	45%	33%	47%	39%	55%	45%	56%	56%^b^	59%^b^	55%
Liver	Top 1	71%	61%	63%	52%	70%	59%	70%	58%	76%^b^	62%	75%	65%^b^
	MV@3	73%	59%	69%	50%	75%	68%	76%	68%	79%^b^	67%	77%	69%^b^
	MV@5	70%	54%	72%	56%	74%	64%	73%	59%	80%	65%	81%^b^	74%^b^
Skin	Top 1	59%	54%	68%	62%	68%	61%	72%	63%	78%	70%	79%^b^	71%^b^
	MV@3	66%	58%	73%	65%	73%	62%	77%	65%	81%	70%	80%^b^	68%^b^
	MV@5	68%	58%	77%	67%	76%	65%	80%	67%	82%^b^	70%^b^	80%	66%
CRC	Top 1	52%	51%	57%	54%	55%	54%	60%^b^	59%	60%^b^	60%^b^	60%^b^	60%^b^
	MV@3	52%	49%	59%	55%	60%	58%	61%^b^	60%	60%	61%^b^	61%^b^	61%^b^
	MV@5	55%	51%	56%	50%	59%	57%	62%	61%	60%	60%	63%^b^	63%^b^
Public Data													
PANDA	Top 1	35%	31%	35%	32%	33%	31%	53%	53%	58%^b^	59%^b^	48%	47%
	MV@3	33%	30%	37%	33%	36%	32%	53%	52%	58%^b^	58%^b^	50%	48%
	MV@5	35%	31%	39%	35%	38%	33%	53%	51%	56%^b^	55%^b^	51%	48%
CAMELYON16	Top 1	57%	56%	66%	65%	60%	59%	70%	68%	75%^b^	72%^b^	62%	57%
	MV@3	58%	56%	66%	64%	58%	55%	71%	68%	72%	68%	74%^b^	69%^b^
	MV@5	57%	54%	64%	62%	64%	61%	70%	65%	79%^b^	75%^b^	75%	70%
BRACS	Top 1	53%	48%	53%	47%	56%	48%	62%	56%	66%^b^	59%^b^	62%	54%
	MV@3	55%	48%	58%	51%	58%	49%	64%	59%^b^	66%^b^	59%^b^	61%	53%
	MV@5	58%	50%	59%	53%	59%	49%	66%	60%^b^	67%^b^	58%	62%	53%

a The Yottixel search engine and leave-1-WSI-out method has been used.

b Best results for Acc and Macro.

In the patch-level search, Table 2 presents a performance comparison of the same models for patch-level retrieval. The evaluation metric is accuracy (Acc) and macro-averaged F1 score (Macro), with a majority vote among top-5 (MV@5).

**Table 2:** Patch Search Results Across 4 Internal and 7 Public Datasets^a^

Dataset	Pretrained on Natural Images				Pretrained on Twitter/PubMed				Pretrained on TCGA			
	DinoV2		CLIP		BiomedCLIP		PLIP		KimiaNet		DinoSSLPath	
	Acc	Macro	Acc	Macro	Acc	Macro	Acc	Macro	Acc	Macro	Acc	Macro
Internal												
Breast	36%	24%	39%	25%	34%	24%	43%	32%	50%^b^	37%^b^	50%^b^	37%^b^
Liver	70%	54%	67%	57%	68%	57%	71%	60%	77%	68%	78%^b^	70%^b^
Skin	68%	58%	68%	60%	66%	58%	71%	63%	73%^b^	65%^b^	72%	63%
CRC	55%	51%	56%	53%	52%	50%	56%	54%	58%	57%^b^	59%^b^	57%^b^
Public												
PANDA	32%	28%	35%	30%	34%	29%	44%	41%	48%^b^	46%^b^	45%	35%
BRACS	51%	44%	53%	46%	51%	43%	56%	50%^b^	38%	28%	57%^b^	50%^b^
CAMELYON16	58%	56%	62%	61%	59%	58%	69%	67%	73%^b^	72%^b^	68%	66%
DigestPath CTS	87%	86%	85%	84%	88%	87%	88%	87%	90%	89%	92%^b^	91%^b^
DigestPath SRCC	96%	93%	94%	91%	97%	95%	98%	96%	99%^b^	98%	99%^b^	99%^b^

a The leave-one-WSI-out method was used, excluding patches sharing the same query.

b Best results for all models are highlighted in green. As Both KimiaNet and DinoSSLPath have been trained with TCGA data, they were excluded from the last 5 datasets. Shows best results for Acc and Macro.

In internal datasets, model performance varies across cancer types. For breast cancer, KimiaNet and DinoSSLPath both reach a macro-averaged F1 score of 37%, denoting a similar balanced classification. In the liver dataset, DinoSSLPath narrowly outperforms other models with scores of 70%, highlighting its proficiency in liver cancer histology. In skin cancer, KimiaNet leads with a 65% F1 score, closely trailed by DinoSSLPath and PLIP, each at 63%. Finally, in the colorectal cancer dataset, DinoSSLPath and KimiaNet equally excel with a 57% F1 score, indicating their suitability for CRC histopathological analysis.

In public datasets, the performance metrics differ widely. In PANDA, KimiaNet leads with a 46% macro-averaged F1 score, significantly outpacing DinoSSLPath’s 35%. For CAMELYON16, KimiaNet again dominates with 72%, whereas DinoSSLPath follows at 66%. In the DigestPath datasets, both colonoscopy tissue segmentation (CTS) and signet ring cell carcinoma (SRCC), models excel across the board. Notably, DinoSSLPath registers 91% and 99% macro-averaged F1 Scores in CTS and SRCC, respectively. KimiaNet follows closely, scoring 89% and 98%.

In the patch-level classification, Table 3 reports the performance of patch classification. In internal datasets, KimiaNet leads in breast and liver with an accuracy of 94.33% and 91.77%, respectively, whereas DinoSSLPath trails at 91.50% and 88.27%, respectively. DinoV2 and CLIP lag, averaging below 80% in these categories. In skin and CRC, KimiaNet again excels with 86.59% and 81.15%, respectively followed by DinoSSLPath at 79.62% and 73.08%, respectively. CLIP and DinoV2 yield moderate results, falling below 76%.

**Table 3:** Quantitative Assessment by 5-Fold Cross-Validation: Performance Metrics Including Accuracy and Macro-Averaged F1 Score for Patch-Level Classification in Histopathological Image Analysis

Dataset	Pretrained on Natural Images				Pretrained on Twitter/PubMed				Pretrained on TCGA			
	DinoV2		CLIP		BiomedCLIP		PLIP		KimiaNet		DinoSSLPath	
	Acc	Macro	Acc	Macro	Acc	Macro	Acc	Macro	Acc	Macro	Acc	Macro
Internal datasets												
Internal	71.2±2.1	59.8±3.1	75.0±1.7	63.3±2.3	59.0±1.1	34.3±1.8	65.1±1.0	38.0±1.9	94.3±0.8^a^	93.6±1.3^a^	91.5±0.8	89.6±1.9
Liver	75.6±12.7	54.4±4.1	83.2±1.1	58.6±3.6	77.4±0.9	49.2±0.9	81.3±1.5	52.4±1.5	91.8±2.3^a^	86.6±2.6^a^	88.3±1.0	80.3±3.8
Skin	73.3±1.5	70.9±2.3	75.6±0.7	72.7±2.0	68.7±0.3	64.1±0.1	73.9±0.3	70.1±0.3	86.6±0.5^a^	86.4±0.6^a^	79.6±1.6	78.1±0.8
CRC	68.7±1.9	63.8±2.3	72.8±1.2	67.5±3.0	65.0±0.2	57.1±1.1	70.6±0.7	62.5±1.9	81.2±1.3^a^	78.0±2.2^a^	73.1±2.2	71.0±2.1
Public datasets												
PANDA	37.7±2.3	27.0±2.0	40.4±2.9	32.5±2.3	39.3±0.5	28.2±0.9	43.0±0.4^a^	32.5±0.7	43.0±3.2	37.6±3.3^a^	39.9±0.5	32.3±3.0
CAMELYON16	62.5±7.3	60.2±7.0	75.0±3.9	73.8±4.8	66.7±2.3	64.3±4.4	75.7±1.4	74.1±2.2	82.2±2.6^a^	81.8±2.5^a^	77.5±2.1	76.6±2.9
BRACS	89.0 ±2.5	88.2 ±2.9	85.2±3.0	84.4±3.5	90.4±2.0	89.5±2.5	86.6±5.3	85.7±5.1	63.4±5.0^a^	57.4±1.5^a^	55.3±9.4	48.6±6.1
DigestPath CTS	89.0±2.5	88.2±2.9	85.2±3.0	84.4±3.5	90.4±2.0	89.5±2.5	86.6±5.3	85.7±5.1	86.3±5.4	85.3 ±6.4	92.9±1.6^a^	92.3±1.8^a^
DigestPath SRCC	95.4±1.4	91.6±3.4	93.8±5.3	89.7±5.7	97.1±1.0	94.7±2.2	96.9±0.9	94.4±1.5	98.0±1.4	96.2±3.3	99.3±0.6^a^	98.8±1.2^a^

a The best Acc and Macro results.

In public datasets, KimiaNet and DinoSSLPath often emerge as leaders. Specifically, KimiaNet leads in PANDA at 43.03% and CAMELYON16 at 82.19%, whereas DinoSSLPath excels in DigestPath CTS and SRCC with 92.90% and 99.34%, respectively. DinoV2 and CLIP generally underperform or show moderate outcomes across these datasets. Figure 3(B) visualizes the classification performance on the public and internal datasets.

## Discussion

Our investigation offers a rigorous evaluation of FMs in histopathology image analysis, highlighting the strengths and weaknesses in their applicability to medical imaging. Notably, while models like DinoSSLPath reported better results on breast and liver datasets with F1 scores up to 65%, their counterparts like KimiaNet provides better results on skin and CRC datasets, achieving F1 scores as high as 70%. This differential performance underscores the challenge of applying FMs, especially those like CLIP and its derivatives, to specialized medical contexts. The limitations often stem from the inability of these generalized models to robustly adapt to the specific needs of medical imaging, particularly when they are not fine-tuned on high-quality, domain-specific datasets.

The role of data quality in influencing model performance is evident. KimiaNet and DinoSSLPath, which were trained on the diverse and (at least comparably) high-quality TCGA dataset, notably outperformed FMs trained with both natural and medical images from internet sources. This underscores the importance of not only quantity but also the quality and relevance of training data. In comparison, FMs like PLIP and BiomedCLIP, reliant on internet-sourced data, fared less effectively, particularly in patch-level retrieval for internal datasets (which represent high-quality clinical data). The lackluster performance of these internet-dependent models raises critical questions about the efficacy of using data that may not have been subjected to rigorous quality control measures.

Furthermore, our study illuminates the performance of domain-specific models. For example, KimiaNet notched an F1 score as high as 75% on the CAMELYON16 dataset, a contrast to the lower scores achieved by more generalized FMs. This disparity underscores the need for domain-specificity when considering the training and application of reliable FMs in medical contexts.

In addition, our performance metrics lend weight to the necessity for expansive, high-quality datasets in medical imaging. Specifically, the near-perfect F1 score of 99% achieved by DinoSSLPath on the SRCC dataset highlights the critical role that data quality plays in the effectiveness of machine learning models in health care.

## Conclusion

Our results emphasize the imperative for a meticulous approach in curating high-quality, domain-specific datasets to train reliable and robust FMs for healthcare applications. The limitations observed in generalized FMs accentuate the need for focused efforts to tailor these models to the unique requirements of medical image analysis.

## Potential Competing interests

The authors declare no conflicts of interest related to the content of this research article.
