# Supplementary material for: Foundation Models for Histopathology—Fanfare or Flair
Source: Mayo Clin Proc Digit Health. 2024 Mar 5;2(1):165–74. doi: 10.1016/j.mcpdig.2024.02.003 (PMC11975837; doi:10.1016/j.mcpdig.2024.02.003)
Supplement: Supplementary Material [file mmc1.pdf]

(Supplementary File)

Foundation Models for Histopathology – Fanfare or Flair

Saghir Alfasly, Ph.D.[1], Peyman Nejat, M.D.[1], Sobhan Hemati, Ph.D.[1], Jibran Khan[1], Isaiah Lahr[1], Areej Alsaafin, Ph.D.[1], Abubakr Shafique, Ph.D.[1], Nneka Comfere, M.D.[2], Dennis Murphree, Ph.D.[2], Chady Meroueh, M.D.[3], Saba Yasir, M.B.B.S[3], Aaron Mangold, M.D.[4], Lisa Boardman, M.D.[5], Vijay H. Shah, M.D.[5], Joaquin J. Garcia, M.D.[3], and H.R.Tizhoosh, Ph.D.[1]

[1]Department of AI & Informatics, Mayo Clinic, Rochester, MN, USA
[2]Department of Dermatology, Mayo Clinic, Rochester, MN, USA
[3]Department of Laboratory Medicine and Pathology, Mayo Clinic, Rochester, MN, USA
[4]Department of Dermatology, Mayo Clinic, Phoenix, AZ, USA
[5]Comprehensive Cancer Center, Mayo Clinic, Rochester, MN, USA

Contents

1 Data **3**
1.1 Internal Datasets . . . 3
1.2 Public Datasets . . . 3

2 Conclusions Summary **4**
2.1 Do We Have Histology Foundation Models in Biomedicine Yet? . . . 4
2.2 The Importance of Large, Clean Medical Datasets . . . 4
2.3 Recommendations . . . 5

3 Tables **6**
3.1 Table 1: Internal Mayo Datasets' Statistic . . . 6
3.2 Table 2: Public Datasets' Statistic . . . 7
3.3 Table 3: Models' Attributes . . . 7

4 Figures **8**
4.1 Performance on WSI-level search Top-1/MV@5 and 5-fold cross-validation. . . . 8
4.2 Performance on WSI-level search MV@3. . . . 8
4.3 Performance on patch-level search MV@5 - Accuracy. . . . 9

4.4 Performance on patch-level search MV@5 - Macro. 9
4.5 Performance with 5-fold cross-validation - Accuracy. 9
4.6 Performance with 5-fold cross-validation - Macro. 10
4.7 Chord Diagrams of BiomedCLIP, PLIP, KimiaNet, and DinoSSLPath. 11
4.8 Confusion matrices on Mayo CRC Dataset - patch-level majority vote MV@5. 12
4.9 T-SNE - Mayo CRC dataset. 12
4.10 Confusion matrices on Mayo liver dataset - patch-level majority vote MV@5. 12
4.11 T-SNE - Mayo liver dataset. 13
4.12 Confusion matrices on Mayo skin dataset - patch-level majority vote MV@5. 13
4.13 T-SNE - Mayo skin dataset. 13
4.14 T-SNE - Mayo breast dataset. 14
4.15 Confusion matrices on DigestPath-CTS dataset - patch-level majority vote MV@5. 14
4.16 T-SNE - DigestPath-CTS dataset. 15
4.17 Confusion matrices on DigestPath-SRCC dataset - patch-level majority vote MV@5. 15
4.18 T-SNE - DigestPath-SRCC dataset. 16

1 Data

1.1 Internal Datasets

We conducted WSI-level retrieval performance evaluation on four internal datasets from the Mayo Clinic each from a different site: Breast, Liver, Skin, and Colorectal datasets.

- **Breast Epithelial Tumors Dataset**: Breast Epithelial Tumors (73 patients) [16 subtypes: 'Adenoid Cystic Carcinoma', 'Adenomy- oepthelioma', 'Ductal Carcinoma In Situ', 'Ductal Carcinoma In Situ, Columnar Cell Lesions Including Flat Epithelial Atypia, Atypical Ductal Hyperplasia', 'Intraductal Papilloma, Columnar Cell Lesions', 'Invasive Breast Carcinoma of No Special Type', 'Invasive lobular carcinoma', 'Lobular Carcinoma In Situ + Atypical Lobular Hyperplasia', 'Lobular Carcinoma In Situ, Flat Epithelial Atypia, Atypical Lobular Hyperplasia', 'Malignant Adenomyoepithelioma', 'Metaplastic Carcinoma', 'Microglandular Adenosis', 'Microinvasive carcinoma', 'Mucinous Cystadenocarcinoma', 'Normal breast', 'Radial scar complex sclerosing lesion']

- **Fatty Liver Disease Dataset**: We obtained liver biopsy slides from patients diagnosed with either ASH or NASH. The diagnosis of ASH was made based on the chart review and expert opinion on history, clinical presentation, and laboratory studies. Liver biopsies from a cohort of morbidly obese patients undergoing bariatric surgery were used to select cases for the NASH group. All the slides were digitized and associated with the corresponding diagnosis as WSI-level label. In total, 150 WSIs of patients with ASH and 158 WSIs of patients with NASH were included.

- **Cutaneous Squamous Cell Carcinoma Dataset (Skin)**: The skin dataset included a total number of 660 skin tissue WSIs of patients diagnosed with cutaneous squamous cell carcinoma (cSCC). The data was pulled from the internal Mayo Clinic database (REDCap). The dataset included 386 cases of well-differentiated, 100 cases of moderately differentiated, and 67 cases of poorly differentiated cSCC. There were also 107 normal WSIs selected to represent the normal skin tissue as a separate group.

- **Colorectal Polyps Dataset (CRC)**: CRC dataset was collected at Mayo Clinic, and comprises a total of 209 WSIs, primarily focusing on colorectal histopathology. This dataset included three distinct categories established for colorectal pathology, including Cancer Adjacent polyps (CAP), Non-recurrent polyps (POP-NR), and Recurrent polyps (POP-R).

1.2 Public Datasets

We used four public datasets for additional histology image retrieval and classification performance evaluation. Note, since we apply the leave-one-out evaluation method, we used the entire training datasets of DigestPath .

- **PANDA dataset** [1] consisted of 12,625 whole-slide images of hematoxylin and eosin stained prostate biopsies including 10,616 biopsies for development, 393 for tuning, 545 for internal validation, and 1,071 for external validation collected from 6 sites in the Netherlands, Sweden, and the United States. Since we evaluate using leave-one-out method, we use the entire dataset for the WSI-based and patch-level retrieval and classification. Total WSI used in our experiments is 10.349 where we derived 87,451 patches using Yottixel patching method.

- **CAMELYON16 dataset** [2] contains 399 whole-slide images of lymph node sections from breast cancer patients. The slides were collected from two hospitals in the Netherlands and meticulously annotated for metastases under pathologist supervision, using immunohistochemistry when needed. Slides contain macrometastases, micrometastases or isolated tumor cells. We used the test set of CAMELYON16 which contains 129 WSI. The final set of used WSI is 128 processed and patched by Yottixel.

- **BRACS dataset** [3] **contains** 547 whole slide images (WSIs) from 189 patients. The images were scanned at 0.25 μm/pixel magnification. The lesions were annotated by three board-certified pathologists into 7 subtypes: normal (484 RoIs), pathological benign (836 RoIs), usual ductal hyperplasia (517 RoIs), flat epithelial atypia (756 RoIs), atypical ductal hyperplasia (507 RoIs), ductal carcinoma in situ (790 RoIs), and invasive carcinoma (649 RoIs). We used the entire dataset since we apply leave-one-out validation. In our experiments, Yottixel processed 523 when patching tissue threshold set to 70% and 5% of the patches are selected. Overall, We used the WSI-level for the patient-level retrieval and we used ROIs for patch-level retrieval and classification.

- **DigestPath** [4]**:** DigestPath contains two datasets for diagnosing digestive-system pathology: the Signet Ring Cell Detection Dataset (SRC) and the Colonoscopy Tissue Segmentation and Classification Dataset (TSCC). They collaborated with esteemed medical institutions in China for curation. The patient demographic includes ages 20 to 70 and a balanced gender distribution. Slides were stained with hematoxylin and eosin (H&E) and scanned using the KFBIO FK-Pro-120 slide scanner. Annotations were meticulously conducted by pathologists. The datasets address Signet Ring Cell Carcinoma detection and colonoscopy tissue segmentation, offering comprehensive resources for pathology research.

2 Conclusions Summary

2.1 Do We Have Histology Foundation Models in Biomedicine Yet?

The quest for foundation models (FMs) in biomedicine has brought us to a critical juncture where we must assess our progress and acknowledge the challenges that still lie ahead. While FMs trained on massive image-text data have demonstrated remarkable capabilities in generalization and adaptability, the question remains: Do we truly have foundation models in biomedicine?

As we have explored in the preceding sections, several critical challenges hinder the development of FMs that can serve as true cornerstones in the medical domain. These challenges span from data quality and size limitations to the complexities of generalization, domain specificity, and the intricacies of fine-tuning. These issues highlight the necessity of large, clean medical datasets for the realization of FMs tailored to the unique demands of biomedicine.

2.2 The Importance of Large, Clean Medical Datasets

The cornerstone of any foundation model is the data it is trained on. In the medical domain, where decisions have life-altering consequences, the quality and quantity of training data are of paramount importance. The lack of diverse and extensive medical datasets not only limits the performance of FMs but also compromises their reliability and trustworthiness.

Additionally, while the capacity for unsupervised learning is evident in machine learning models, the medical domain demands a different approach, necessitating the integration of human expertise. Even in the domain of natural image processing, the success of ChatGPT has been notably influenced by the final phase of its training, which heavily relies on Reinforcement Learning from Human Feedback (RLHF) [5]. The preparation of this feedback involves substantial efforts by OpenAI's dedicated team of experts. However, it is important to acknowledge that OpenAI continually seeks to enhance ChatGPT's performance by incorporating user feedback, which may not consistently adhere to ideal standards of quality and optimization. As a result, there have been observations of ChatGPT generating responses of lower quality [6]. Large, clean medical datasets, meticulously curated and validated by experts, are essential for capturing the diversity of medical conditions, nuances, and variations that exist in the real world. These datasets enable FMs to learn and generalize effectively, bridging the gap between research and practical medical applications.

The Current State of Biomedical Foundation Models. Currently, we are at a juncture where FMs fine-tuned on limited, noisy, or non-representative medical datasets may not truly be considered foundation models. While these models may offer valuable insights and assistance, they fall short of the robustness, accuracy, and generalization capabilities expected of foundation models.

Moreover, the contrast becomes evident when we compare these fine-tuned FMs with modality-specific models, trained on clean and comprehensive datasets, which often outperform them in some medical image analysis tasks. This performance gap underscores the pressing need for a paradigm shift in the development of biomedicine-specific foundation models.

All in all, while foundation models have shown tremendous promise in various domains, the journey to establishing them in biomedicine is far from complete. As long as we continue to rely on small, noisy, or non-representative datasets, we will fall short of achieving true foundation models for the medical domain. The path forward necessitates a concerted effort to build and curate large, clean medical datasets, paving the way for foundation models that can usher in transformative advancements in healthcare and biomedicine.

2.3 Recommendations

The Path Forward Building True Biomedical Foundation Models:

- To establish true foundation models in biomedicine, the foremost imperative is the creation of large, clean medical datasets. These datasets should be extensively validated by multiple experts, and encompass a broad spectrum of medical conditions, imaging modalities, and textual data.
- Efforts should focus on enhancing collaboration among experts, institutions, and the AI research community to ensure the availability and accessibility of such datasets. Ethical considerations, data privacy, and regulatory compliance must also be carefully addressed in this process.

3 Tables

3.1 Table 1: Internal Mayo Datasets' Statistic

Table 1: Internal histology image datasets. Four different datasets were collected at Mayo Clinic for four sites including Liver, Skin, Breast, and colon sites.

Dataset	#Class	#WSI	#Patches	Diagnosis Acronyms	Diagnosis
				CAP	Cancer Adjacent polyp
Colorectal	3	209	4,619	POP-NR	Non-recurrent polyp
				POP-R	Recurrent polyp
				ASH	Alcoholic Steatohepatitis
Liver	3	324	2,976	NASH	Non-alcoholic Steatohepatitis
				Normal	Normal tissue
				Normal	Normal skin
Skin	4	660	8,390	ModDiff	Moderately differentiated
				PoorDiff	Poorly differentiated
				WellDiff	Well differentiated
				ACC	Adenoid Cystic Carcinoma
				AME	Adenomyoepthelioma
				DCIS	Ductal Carcinoma In Situ
				DCIS_CCL_FEA_ADH	Ductal Carcinoma In Situ,
				Columnar Cell Lesions Including Flat	
					Epithelial Atypia, Atypical Ductal Hyperplasia
				IP_CCL	Intraductal Papilloma, Columnar Cell Lesions
Breast	16	73	1,141	IBC	Invasive Breast Carcinoma of No Special Type
				ILC	Invasive lobular carcinoma
				LCIS_ALH	Lobular Carcinoma In Situ +
				Atypical Lobular Hyperplasia	
				LCIS_FEA_ALH	Lobular Carcinoma In Situ,
				Flat Epithelial Atypia,	
				Atypical Lobular Hyperplasia	
				MAME	Malignant Adenomyoepithelioma
				MC	Metaplastic Carcinoma
				MA	Microglandular Adenosis
				MIC	Microinvasive carcinoma
				MCC	Mucinous Cystadenocarcinoma
				RSCSL	Radial scar complex sclerosing lesion
				Normal	Normal breast

3.2 Table 2: Public Datasets' Statistic

Table 2: Public histology image datasets including PANDA, CAMELYON16, BRACS, and DigestPath. Number of images represents the total images (patches) used in the evaluation regardless its training/testing split since we used the leave-one-out evaluation method for the search task and k-fold cross-validation for the classification task.

Dataset	Analysis Scale	#Class	#WSI	#Image	Acronyms	Diagnosis
PANDA	WSI/Patch	6	10,349	87,451	0	ISUP-grading 0
					1	ISUP-grading 1
					2	ISUP-grading 2
					3	ISUP-grading 3
					4	ISUP-grading 4
					5	ISUP-grading 5
CAMELYON16	WSI/Patch	2	128	2,864	Tumor	Tumor
					Normal	Normal
BRACS	WSI/Patch	3	523	10,984	Group-BT	Benign
					Group-AT	Atypical
					Group-MT	Malignant
DigestPath	Patch-Level	2	-	1,103	neg	Benign
					pos	Malignant

3.3 Table 3: Models' Attributes

Table 3: Summary of Model Attributes. Note, DinoV2, CLIP, and DinoSSLPath trained CNN-based backbones such as ResNet50, however, we only employee the Transformer-based backbones in our comparisons. Floating Point Operations Per Second (FLOPs) used to quantify the computational complexity of models.

Model	Pretrained On	Pretraining Domain	Modality	Data Size	Structure	Learning Paradigm	Input Size	Model Params	FLOPs
DinoV2 [7]	Internet	Natural Images	Image	142 million	Transformer	Self-Suppervised	224×224	$85,508,352$	$21,963,549,696$
CLIP [8]	Internet	Natural Images	Image-Text	400 million	Transformer	Contrastive Learn.	224×224	$85,646,592$	$16,862,862,336$
BiomedCLIP [9]	PMC-15M	Medical (PubMed)	Image-Text	13.9 million	Transformer	Contrastive Learn.	224×224	$85,646,592$	$16,862,862,336$
PLIP [10]	OpenPath	Histology (Twitter)	Image-Text	$208,414$	Transformer	Contrastive Learn.	224×224	$85,646,592$	$16,862,862,336$
KimiaNet [11]	TCGA	Histology Images	Image	$240,000$	CNN	Suppervised	1000×1000	$6,953,856$	$57,471,584,640$
DinoSSLPath [12]	TCGA	Histology Images	Image	19 million	Transformer	Self-supervised	224×224	$21,368,448$	$16,756,372,992$

4 Figures

4.1 Performance on WSI-level search Top-1/MV@5 and 5-fold cross-validation.

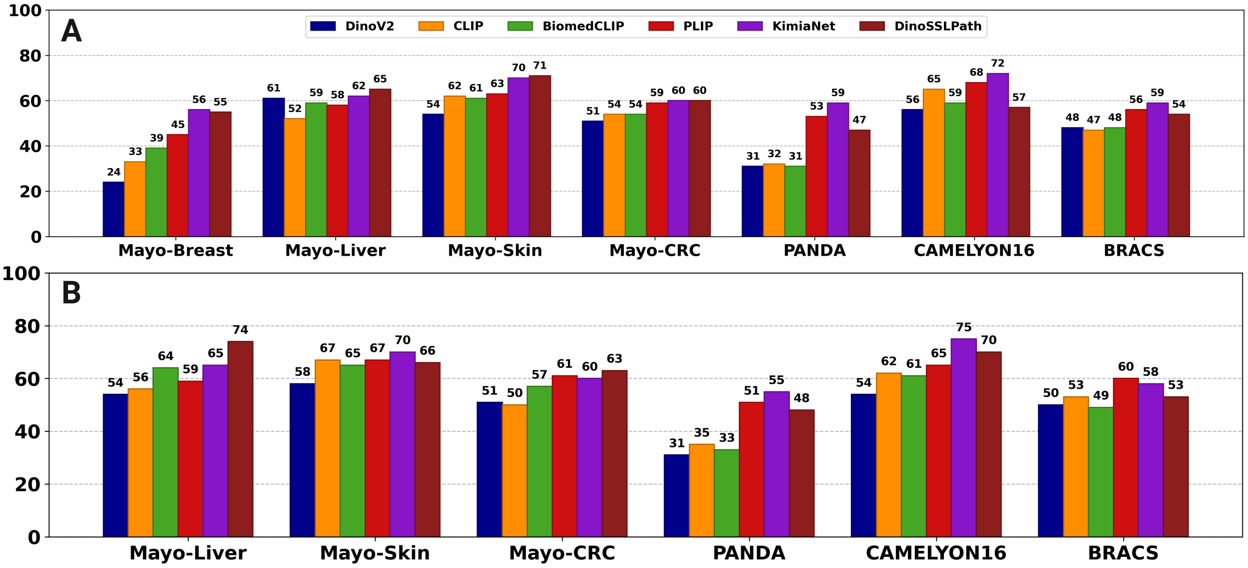

Figure 1: Model Performance on Public and Private Datasets: (A) Top-1 Macro F-Score under WSI-Level Search, (B) MV@5 Majority Vote Performance under WSI-Level Search.

4.2 Performance on WSI-level search MV@3.

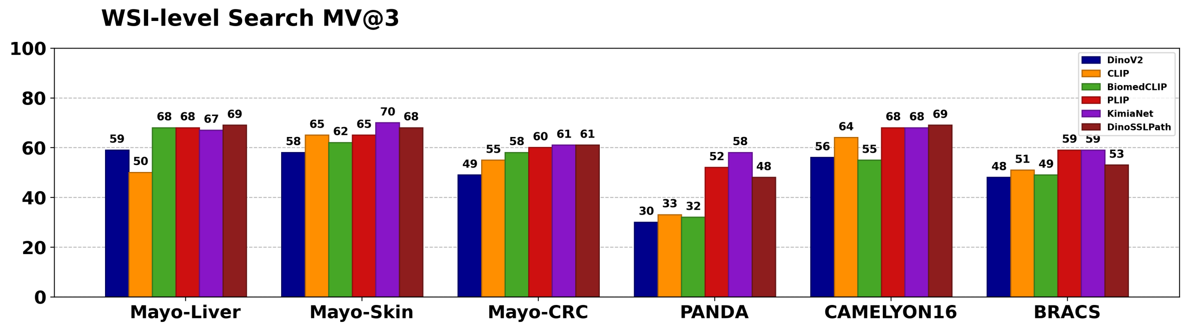

Figure 2: Performance of DinoV2, CLIP, BiomedCLIP, PLIP, DinoSSLPath, and KimiaNet models on several datasets with WSI-level settings and Majority Vote MV@3 of Macro F-score.

4.3 Performance on patch-level search MV@5 - Accuracy.

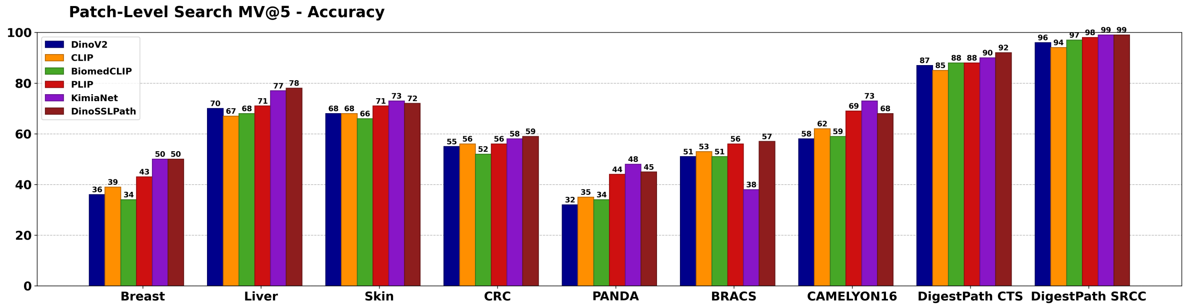

Figure 3: Performance of DinoV2, CLIP, BiomedCLIP, PLIP, DinoSSLPath, and KimiaNet models on several datasets with patch-level settings and Majority Vote MV@5 of **Accuracy** metric.

4.4 Performance on patch-level search MV@5 - Macro.

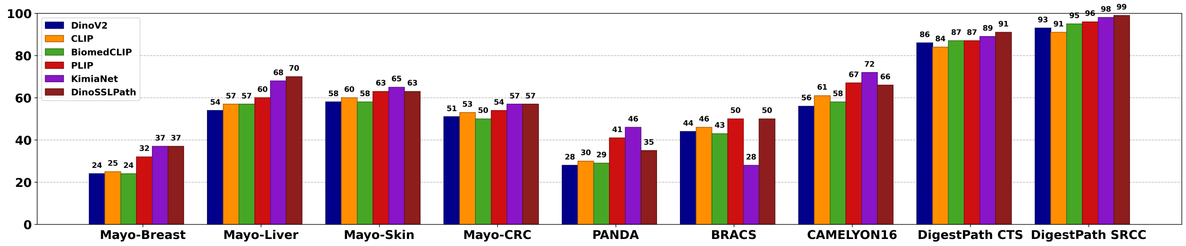

Figure 4: Performance of DinoV2, CLIP, BiomedCLIP, PLIP, DinoSSLPath, and KimiaNet models on several datasets with patch-level settings and Majority Vote MV@5 of **Macro** metric.

4.5 Performance with 5-fold cross-validation - Accuracy.

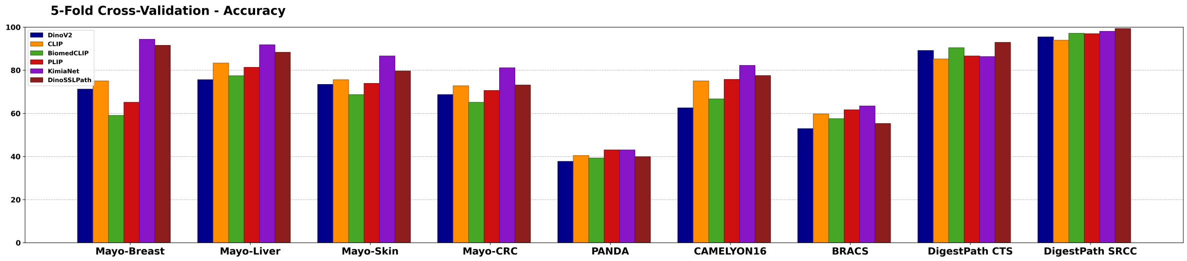

Figure 5: Performance of DinoV2, CLIP, BiomedCLIP, PLIP, DinoSSLPath, and KimiaNet models on several datasets with 5-fold cross-validation and **Accuracy** metric.

4.6 Performance with 5-fold cross-validation - Macro.

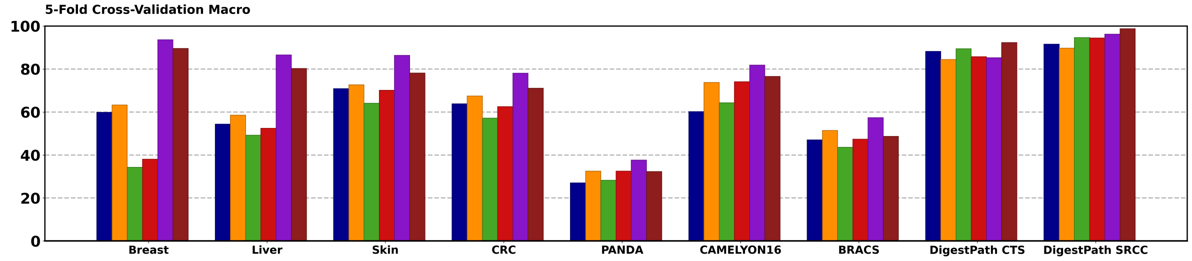

Figure 6: Performance of DinoV2, CLIP, BiomedCLIP, PLIP, DinoSSLPath, and KimiaNet models on several datasets with 5-fold cross-validation and **Macro Avg F-score** metric.

4.7 Chord Diagrams of BiomedCLIP, PLIP, KimiaNet, and DinoSSLPath.

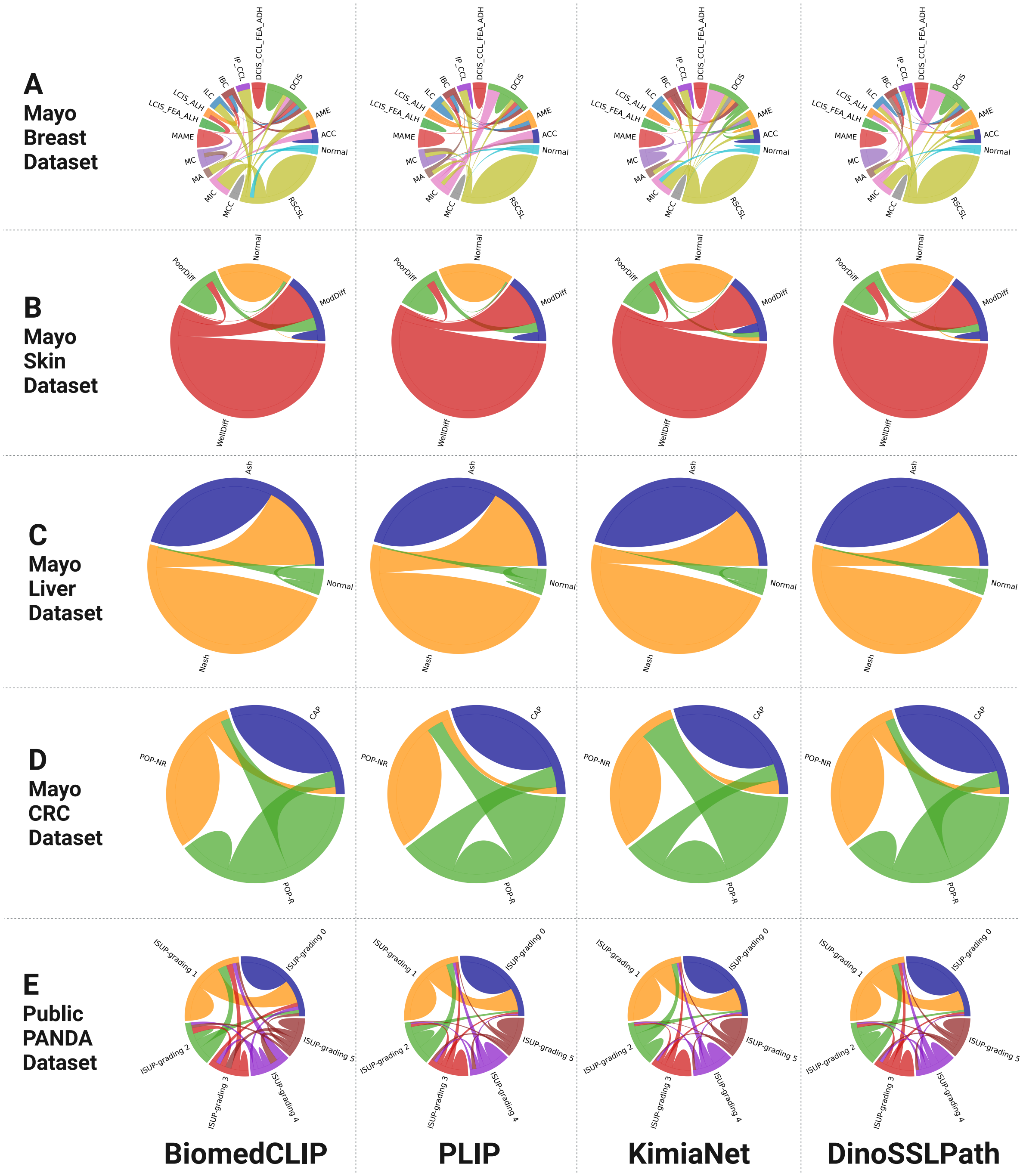

Figure 7: Chord Diagrams of BiomedCLIP, PLIP, KimiaNet, and DinoSSLPath on five datasets: (A) Mayo breast dataset, (B) Mayo skin dataset, (C) Mayo liver dataset, (D) Mayo CRC dataset, and (E) public PANDA dataset.

4.8 Confusion matrices on Mayo CRC Dataset - patch-level majority vote MV@5.

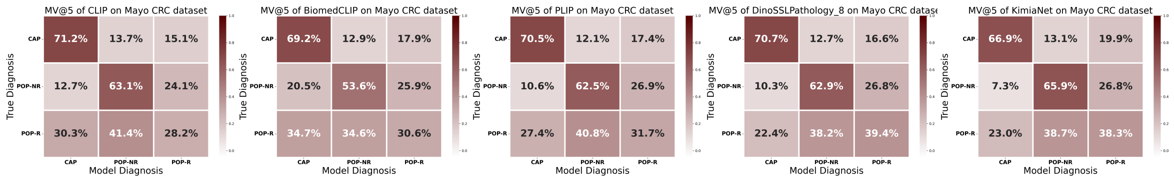

Figure 8: Performance of CLIP, BiomedCLIP, PLIP, DinoSSLPath, and KimiaNet models on Mayo colorectal Dataset with patch-level settings and Majority Vote MV@5 metric.

4.9 T-SNE - Mayo CRC dataset.

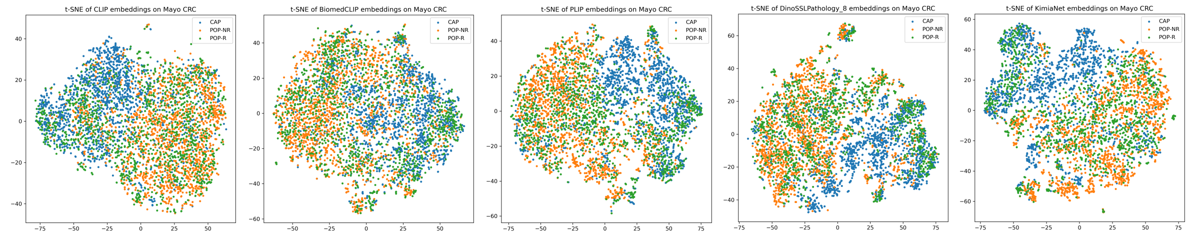

Figure 9: T-SNE visualizes the feature embeddings of CLIP, BiomedCLIP, PLIP, DinoSSLPath, and KimiaNet models on Mayo colorectal dataset with patch-level settings.

4.10 Confusion matrices on Mayo liver dataset - patch-level majority vote MV@5.

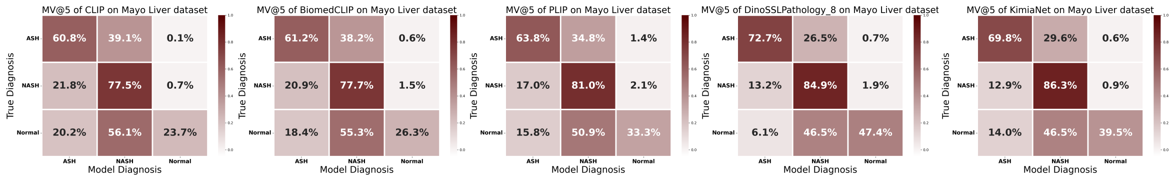

Figure 10: Performance of CLIP, BiomedCLIP, PLIP, DinoSSLPath, and KimiaNet models on Mayo liver dataset with patch-level settings and Majority Vote MV@5 metric.

4.11 T-SNE - Mayo liver dataset.

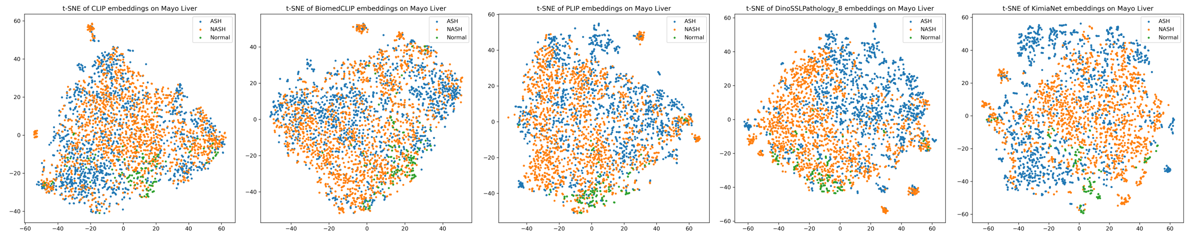

Figure 11: T-SNE visualizes the feature embeddings of CLIP, BiomedCLIP, PLIP, DinoSSLPath, and KimiaNet models on Mayo liver dataset with patch-level settings.

4.12 Confusion matrices on Mayo skin dataset - patch-level majority vote MV@5.

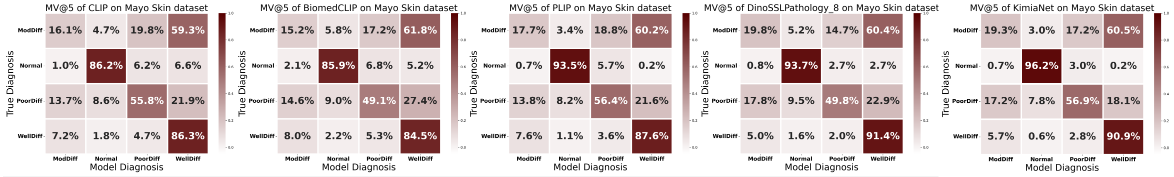

Figure 12: Performance of CLIP, BiomedCLIP, PLIP, DinoSSLPath, and KimiaNet models on Mayo skin dataset with patch-level settings and Majority Vote MV@5 metric.

4.13 T-SNE - Mayo skin dataset.

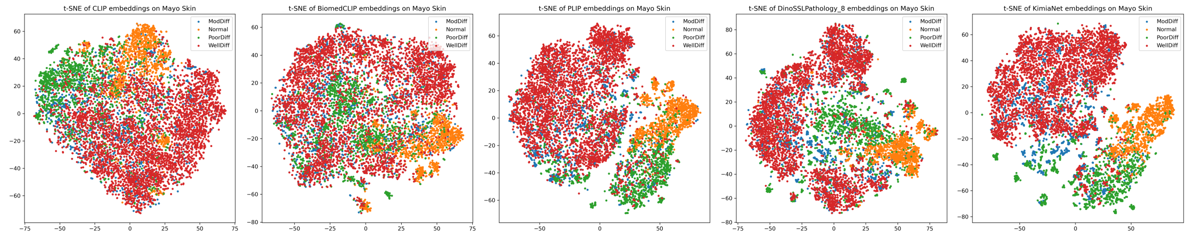

Figure 13: T-SNE visualizes the feature embeddings of CLIP, BiomedCLIP, PLIP, DinoSSLPath, and KimiaNet models on Mayo skin dataset with patch-level settings.

4.14 T-SNE - Mayo breast dataset.

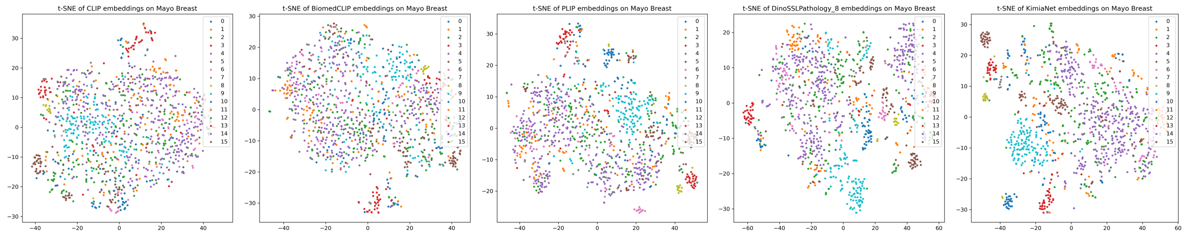

Figure 14: T-SNE visualizes the feature embeddings of CLIP, BiomedCLIP, PLIP, DinoSSLPath, and KimiaNet models on Mayo breast dataset with patch-level settings.

4.15 Confusion matrices on DigestPath-CTS dataset - patch-level majority vote MV@5.

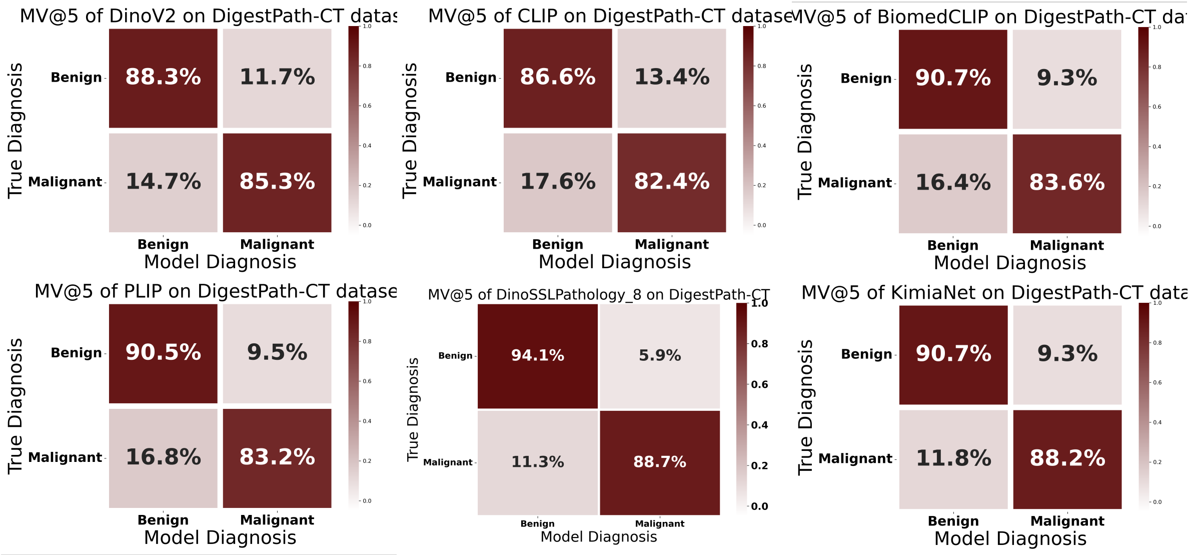

Figure 15: Performance of DinoV2, CLIP, BiomedCLIP, PLIP, DinoSSLPath, and KimiaNet models on DigestPath CT dataset.

4.16 T-SNE - DigestPath-CTS dataset.

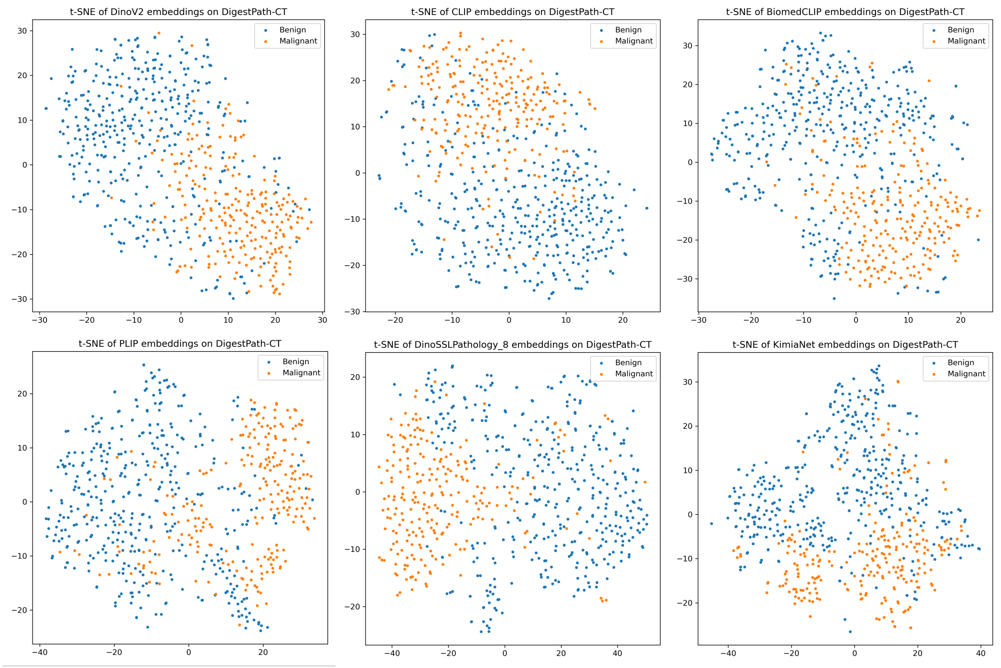

Figure 16: T-SNE visualizes the feature embeddings of DinoV2, CLIP, BiomedCLIP, PLIP, DinoSSLPath, and KimiaNet models on DigestPath CT dataset.

4.17 Confusion matrices on DigestPath-SRCC dataset - patch-level majority vote MV@5.

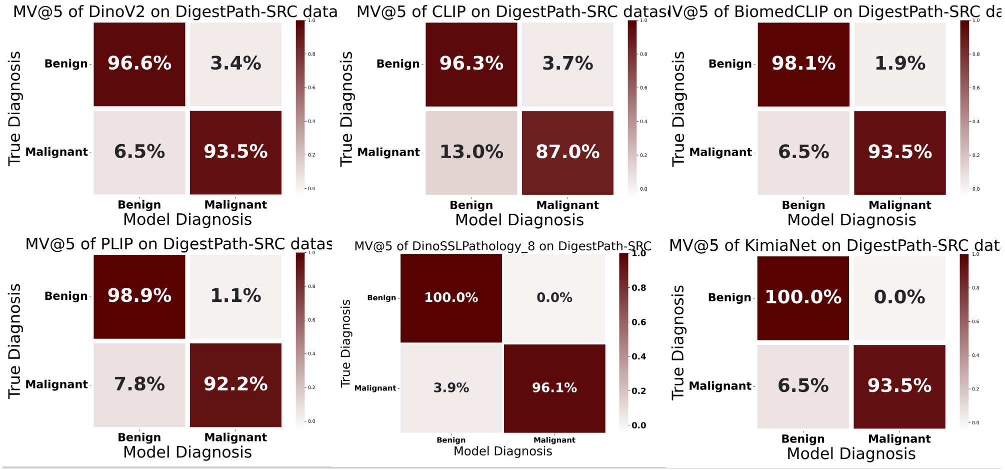

Figure 17: Performance of DinoV2, CLIP, BiomedCLIP, PLIP, DinoSSLPath, and KimiaNet models on DigestPath SRC dataset.

4.18 T-SNE - DigestPath-SRCC dataset.

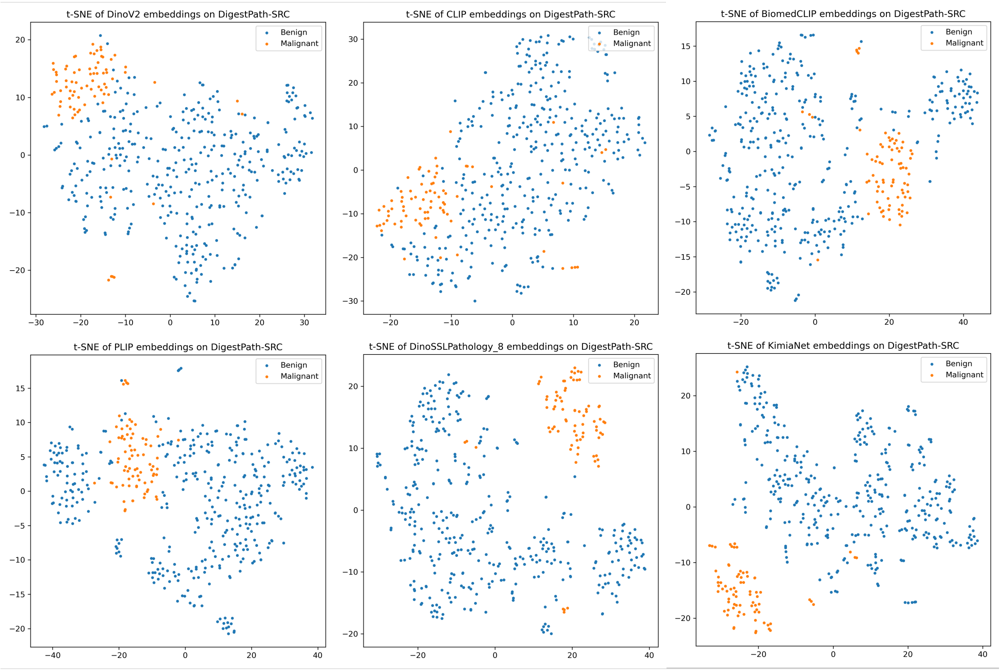

Figure 18: T-SNE visualizes the feature embeddings of DinoV2, CLIP, BiomedCLIP, PLIP, DinoSSLPath, and KimiaNet models on DigestPath SRC dataset.

References

[1] Wouter Bulten, Kimmo Kartasalo, Po-Hsuan Cameron Chen, Peter Ström, Hans Pinckaers, Kunal Nagpal, Yuannan Cai, David F Steiner, Hester van Boven, Robert Vink, et al. Artificial intelligence for diagnosis and gleason grading of prostate cancer: the panda challenge. *Nature medicine*, 28(1):154–163, 2022.

[2] Babak Ehteshami Bejnordi, Mitko Veta, Paul Johannes Van Diest, Bram Van Ginneken, Nico Karssemeijer, Geert Litjens, Jeroen AWM Van Der Laak, Meyke Hermsen, Quirine F Manson, Maschenka Balkenhol, et al. Diagnostic assessment of deep learning algorithms for detection of lymph node metastases in women with breast cancer. *Jama*, 318(22):2199–2210, 2017.

[3] Nadia Brancati, Anna Maria Anniciello, Pushpak Pati, Daniel Riccio, Giosuè Scognamiglio, Guillaume Jaume, Giuseppe De Pietro, Maurizio Di Bonito, Antonio Foncubierta, Gerardo Botti, Maria Gabrani, Florinda Feroce, and Maria Frucci. BRACS: A Dataset for BReAst Carcinoma Subtyping in H amp;E Histology Images. *Database*, 2022:baac093, 10 2022.

[4] Qian Da, Xiaodi Huang, Zhongyu Li, Yanfei Zuo, Chenbin Zhang, Jingxin Liu, Wen Chen, Jiahui Li, Dou Xu, Zhiqiang Hu, et al. Digestpath: A benchmark dataset with challenge review for the pathological detection and segmentation of digestive-system. *Medical Image Analysis*, 80:102485, 2022.

[5] Nisan Stiennon, Long Ouyang, Jeffrey Wu, Daniel Ziegler, Ryan Lowe, Chelsea Voss, Alec Radford, Dario Amodei, and Paul F Christiano. Learning to summarize with human feedback. *Advances in Neural Information Processing Systems*, 33:3008–3021, 2020.

[6] Lingjiao Chen, Matei Zaharia, and James Zou. How is chatgpt's behavior changing over time? *arXiv preprint arXiv:2307.09009*, 2023.

[7] Maxime Oquab, Timothée Darcet, Théo Moutakanni, Huy Vo, Marc Szafraniec, Vasil Khalidov, Pierre Fernandez, Daniel Haziza, Francisco Massa, Alaaeldin El-Nouby, et al. Dinov2: Learning robust visual features without supervision. *arXiv preprint arXiv:2304.07193*, 2023.

[8] Alec Radford, Jong Wook Kim, Chris Hallacy, Aditya Ramesh, Gabriel Goh, Sandhini Agarwal, Girish Sastry, et al. Learning transferable visual models from natural language supervision. In *International conference on machine learning*, pages 8748–8763. PMLR, 2021.

[9] Sheng Zhang, Yanbo Xu, Naoto Usuyama, Jaspreet Bagga, Robert Tinn, Sam Preston, Rajesh Rao, et al. Large-scale domain-specific pretraining for biomedical vision-language processing. *arXiv preprint arXiv:2303.00915*, 2023.

[10] Zhi Huang, Federico Bianchi, Mert Yuksekgonul, Thomas J Montine, and James Zou. A visual–language foundation model for pathology image analysis using medical twitter. *Nature Medicine*, pages 1–10, 2023.

[11] Abtin Riasatian, Morteza Babaie, Danial Maleki, Shivam Kalra, Mojtaba Valipour, Sobhan Hemati, Manit Zaveri, et al. Fine-tuning and training of densenet for histopathology image representation using tcga diagnostic slides. *Medical Image Analysis*, 70:102032, 2021.

[12] Mingu Kang, Heon Song, Seonwook Park, Donggeun Yoo, and Sérgio Pereira. Benchmarking self-supervised learning on diverse pathology datasets. In *Proceedings of the IEEE/CVF Conference on Computer Vision and Pattern Recognition (CVPR)*, pages 3344–3354, June 2023.
